# Effect of a carrageenan/chitosan coating with allyl isothiocyanate on microbial spoilage and quality of chicken breast

**DOI:** 10.1016/j.psj.2022.102442

**Published:** 2022-12-21

**Authors:** Amanda Moller, Cortney Leone, Jasmine Kataria, Gaganpreet Sidhu, Estefania Novoa Rama, Brenda Kroft, Harshavardhan Thippareddi, Manpreet Singh

**Affiliations:** ⁎ Department of Food Science and Technology, University of Georgia, Athens, GA 30602, USA; † Department of Poultry Science, University of Georgia, Athens, GA 30602, USA

**Keywords:** allyl isothiocyanate, carrageenan, chitosan, chicken breast

## Abstract

Approximately 3.7% of poultry meat is lost due to spoilage each year in the United States. The objective of this study was to determine the efficacy of a layered carrageenan/chitosan coating in combination with an application of two concentrations of allyl isothiocyanate (**AITC**) against lactic acid bacteria, aerobic bacteria, and yeast and mold during storage of chicken breast for 21 d. Additionally, the rancidity, color, and pH of the chicken breast as indicators of non-microbial quality were evaluated. The combination of carrageenan/chitosan coating with 20 and 200 ppm AITC reduced (*P* ≤ 0.05) yeast and mold populations by 3 log_10_ CFU/g at d 21 compared to the untreated control. The carrageenan/chitosan coating with 20 and 200 ppm AITC delayed aerobic spoilage by 3 and 12 d, respectively, compared to the untreated control; aerobic bacteria populations on the samples treated with 200 ppm AITC remained below the threshold for spoilage (∼6 log_10_ CFU/g) for the duration of storage. The pH of the 20 ppm and 200 ppm AITC-treated chicken breast was unaltered (*P* > 0.05) at the end of storage and was lower than the pH of the untreated and coating-only-treated control chicken breast at d 18 through the end of storage (*P* ≤ 0.05). The application of the coating alone did not (*P* > 0.05) affect L*, a*, and b* values of the chicken breast at the end of storage compared to the uncoated control. The carrageenan/chitosan coating with 20 and 200 ppm AITC prevented decreases in the lightness (L* values) of the chicken breast at the end of storage (*P* ≤ 0.05) compared to the control and coating-only-treated samples. The coating alone or with AITC did not (*P* > 0.05) impact the rancidity of the chicken breast over the 21-d storage period, thus showing potential to be used as antimicrobial packaging to increase shelf life of fresh poultry.

## INTRODUCTION

The United States is the world's top poultry producer (USDA, 2021), and poultry consumption in the US has doubled since 1978. Chicken breast is the most frequently consumed cut of poultry meat (USDA, 2018), however, with increased production and consumption, approximately 3.7 to 4.2% of poultry meat is lost because of spoilage each year (USDA, 2009). Spoilage of meat can be attributed to prolonged storage times, improper storage temperatures, contamination, or high pH levels. Furthermore, spoilage is detrimental to product quality because of the development of off-flavors, off-colors, off-odors, and microbial growth (Hinton, 2016). Hence, poultry processors are seeking emerging technologies to reduce spoilage and prevent the associated impact on the quality of poultry meat. While techniques such as heat treatment, salting, and acidification have been applied in the food industry for decades to minimize spoilage (Lucera et al., 2012), edible coatings and films are an alternative emerging technology to increase the shelf life of meat and poultry products.

Edible films and coatings can be composed of proteins, polysaccharides, lipids, or carbohydrates alone or as composites of these compounds. Coatings can control gas exchange, moisture permeation, gas permeation, or oxidation (Han, 2014), and Pinheiro et al. (2012) reported that carrageenan and chitosan are suitable for use as coatings because their opposing electrostatic charges enable the formation of multilayered structures. Chitosan is a cationic polysaccharide obtained from the deacetylation of chitin and possesses oxygen barrier properties as well as antimicrobial and antioxidant activity (Xia et al., 2011). Carrageenan is a sulfated anionic polysaccharide used in the food industry as a gelling and stabilizing agent (Yegappan et al., 2018). Edible coatings can be applied by spraying, dipping, and spreading. Spraying is of interest to food processors because of the potential cost reduction and the high quality of the final product compared to other conventional techniques (Andrade et al., 2012). The droplet size while using sprayers can be as small as 20 µm, increasing the surface area of the coating and forming a coating with an even surface, however, coatings with more than one layer may require multiple sprayers and drying steps. Spreading, also known as brushing, applies coating onto a surface and can be applied to the production of polysaccharides and protein-based films (Thebault and Jouenne, 2013). Spreading can be affected the surface texture, environmental conditions, and liquid properties (Khan and Nasef, 2009). Dipping techniques form thick membranous films over the product surface by directly dipping the product into the aqueous coating formulation and further air-drying, however, the ideal amount of coating solution cannot be easily controlled with the dipping technique. Dipping can also be applied using the layer-by-layer technique, which has proven to be an effective technique due to the possibility of controlling the antimicrobial release and incorporating a wide range of biological functions (Silva-Buzanello et al., 2019). Studies have also shown that the ability of antimicrobials to diffuse across the membrane increases when there is a low interpolation between layers. A layer-by-layer technique is employed to create a 6-layer carrageenan/chitosan coating to which an antimicrobial agent can attach and diffuse (Pinheiro et al., 2012).

Allyl isothiocyanate (**AITC**) is a volatile and aliphatic sulfur-containing antimicrobial compound extracted from mustard seeds (Lin et al., 2000). AITC is a generally recognized as safe (**GRAS**) substance (FDA, 2016) and the primary flavor component in foods such as wasabi, horseradish, and mustard (Corrales et al., 2014). Gaseous AITC can prevent the growth of bacteria and fungi by inducing metabolite leakage and a threefold increase in galactosidase activity (Lin et al., 2000). However, the use of AITC in food systems is limited because of its high volatility and pungent odor. Adding AITC to kimchi has been found to increase pH, reduce titratable acidity, decrease spoilage bacteria, improve texture, and enhance the product's shelf life; however, the overall scores of acceptability were reduced because of the odor of the AITC (Ko et al., 2012). The objective of this study was to develop a carrageenan/chitosan coating used in combination with a 20 or 200 ppm AITC treatment, determine its efficacy against spoilage microbes on chicken breast, and assess the effects on the quality metrics of color, pH, and rancidity.

## MATERIALS AND METHODS

### Preparation of Antimicrobial Coatings and Allyl Isothiocyanate

A 0.2% (w/v) carrageenan (Sigma-Aldrich, St. Louis, MO) coating was prepared by adding 8 g of carrageenan to 4 L of distilled water, and the suspension was mixed on a stir plate for 24 h at room temperature. The 0.2% (w/v) chitosan (Sigma-Aldrich, St. Louis, MO) coating was prepared similarly in 4 L of 1% lactic acid (Fisher Scientific, Fair Lawn, NJ). The coatings were placed in two separate glass containers for immersing the chicken breast samples. An AITC (≥95% purity; Koptec, King of Prussia, PA) working stock solution (1:5 dilution) was prepared by adding 2 mL of AITC to 8 mL of 90-proof ethanol (Fisher Scientific, Fair Lawn, NJ). An additional dilution of this stock was prepared by adding 1 mL of the 1:5 dilution to 9 mL of 90-proof ethanol to give a 1:50 working stock solution. The working stocks were then used to prepare the AITC treatment solutions on the day of the experiment by separately adding 1 mL of each working stock to 999 mL of sterile distilled water to obtain 2 solutions at final AITC concentrations of 20 and 200 ppm.

### Application of Antimicrobial Treatment

Fresh chicken breasts were purchased from a local grocery store and immediately transported to the laboratory. The chicken breasts were aseptically cut into 20 g pieces and divided into two groups: samples for (1) non-microbial quality and (2) microbial spoilage. Each group was subdivided into 4 treatment categories: (1) coating + AITC 20 ppm, (2) coating + AITC 200 ppm, (3) coating only, and (4) no coating. The chicken breast samples with no coating served as a positive control. The remaining samples were immersed in coatings in the sequence of carrageenan-chitosan-carrageenan-chitosan-carrageenan to create a multilayer coating. After each layer was applied, the samples were allowed to dry in a laminar flow biological safety cabinet for 15 min prior to rinsing with sterile distilled water. The AITC treatment was applied following the application of the multilayer carrageenan-chitosan coating, by dipping in 20 or 200 ppm of AITC and allowed to dry for 15 min prior to rinsing with sterile distilled water. All samples were stored at 4°C until analysis.

### Evaluation of Microbial Spoilage

Duplicate chicken breast samples for each treatment group were removed from the incubator at each sampling timepoint (0, 3, 6, 9, 12, 15, 18, and 21 d). The samples were placed in sterile bags and rinsed with 20 mL of buffered peptone water (**BPW**; BD Difco, Sparks, MD). Samples were rinsed for 30 s at 300 rpm in a stomacher (Stomacher400 Circulator, Seward, West Sussex, UK). The rinsate was collected, and 1 mL of the rinsate was serially diluted in 9 mL of phosphate-buffered saline (**PBS**). The appropriate dilutions were plated on Petrifilm lactic acid bacteria (**LAB**) count plates, rapid aerobic count plates, and yeast and mold count plates (3M, St. Paul, MN). The rapid aerobic count plates and LAB count plates were incubated at 37°C for 24 h and 48 h, respectively. The yeast and mold count plates were incubated at 25°C for 72 h.

### Quality Analysis

Duplicate chicken breast samples for each treatment group were removed from the incubator at each sampling timepoint (0, 3, 6, 9, 12, 15, 18, and 21 d). The color on the surface of each sample was measured using a chromameter (CR-400, Konica Minolta, Tokyo, Japan) according to the CIE-L*a*b* system. The pH on the surface of each sample was measured using a surface pH meter (HQ11D, Hach, Loveland, CO).

The rancidity was quantified by blending (Original Magic Bullet, Nutribullet, Los Angeles, CA) 10 g of chicken breast with deionized water for 2 min. Two milliliters of the homogenate was combined with 4 mL of trichloroacetic acid (**TCA**)/thiobarbituric acid (**TBA**) reagent [20 mM TBA (Fisher Scientific, Fair Lawn, NJ), 15% TCA (Sigma-Aldrich, St. Louis, MO)] and 100 µL of 10% butylated hydroxyanisole (in 90% ethanol). The mixture was vortexed thoroughly, heated in a boiling water bath for 15 min, cooled in an ice bath for 10 min, and centrifuged at 2,000 × *g* for 10 min. The supernatant was collected, and the absorbance at 531 nm was measured using a spectrophotometer (Genesys 10S UV-Vis, Thermo Fisher Scientific, Waltham, MA). The absorbance of the reaction supernatant for each sample was compared to a tetraethoxypropane (**TEP**) standard curve constructed using the OD_531_ values of TEP solutions ranging from 0 to 50 µg/mL, from which the concentration of malonaldehyde (µg/g tissue) in the chicken breast sample was determined.

### Statistical Analysis

Three experimental replications were performed on separate days. Fresh carrageenan and chitosan coating solutions and AITC solutions were prepared for each replication, and fresh chicken breasts were purchased on the day of the experiment. The data were analyzed using analysis of variance with the general linear model of SAS (SAS 9.4 Institute, Inc., Cary, NC). Statistical differences between the treatments are reported as least squares means, and significance is reported at *P* ≤ 0.05.

## RESULTS AND DISCUSSION

### Microbial Spoilage

**Figure 1:**
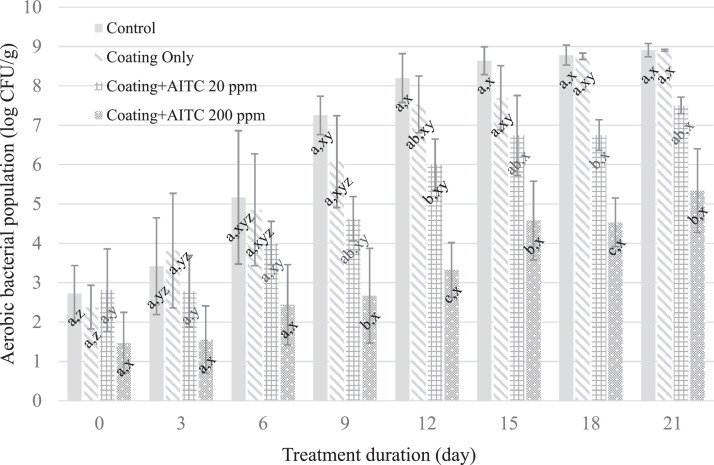
Mean aerobic bacteria populations on chicken breast treated with no coating, coating only, coating + 20 ppm AITC, and coating + 200 ppm AITC. A–C: Different means within a timepoint are indicated by different superscript letters (*P* ≤ 0.05). x–z: Different means within a treatment condition are indicated by different superscript letters (*P* ≤ 0.05).

**Figure 2:**
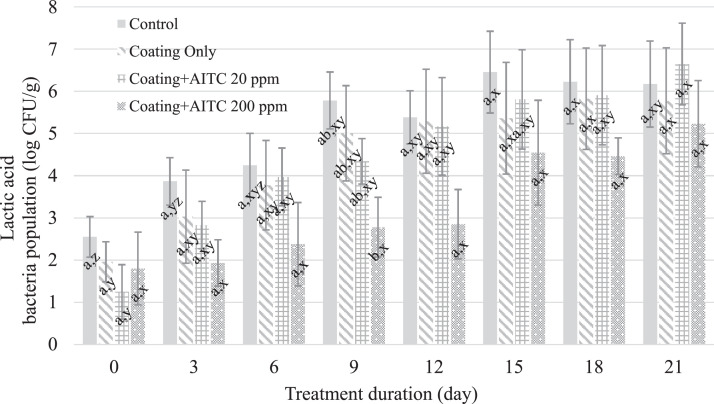
Mean lactic acid bacteria populations on chicken breasts treated with no coating, coating only, coating + 20 ppm AITC, and coating + 200 ppm AITC. A–B: Different means within a timepoint are indicated by different superscript letters (*P* ≤ 0.05). X–Z: Different means within a treatment condition are indicated by different superscript letters (*P* ≤ 0.05).

Aerobic bacteria spoilage occurs on meats at a pH greater than 6.0 and when the aerobic bacterial populations reach 6 log_10_ CFU/cm^2^; LAB spoilage, under anaerobic conditions, occurs above a bacterial population of 8 log_10_ CFU/cm^2^, but such spoilage can be altered by the presence of oxygen (Gill, 2003). In this study, the aerobic bacteria populations on the chicken breast increased (P ≤ 0.05) for the untreated control, coating-only, and coating + AITC 20 ppm treatments over the 21-d storage period at 4°C (Figure 1). Chicken breast without coating and coating only reached aerobic spoilage by d 9; in contrast, chicken breast treated with coating + AITC 20 ppm reached aerobic spoilage by d 12. The aerobic bacteria populations on chicken breast treated with coating + AITC 200 ppm were lower (*P* ≤ 0.05) than the untreated control, coating-only-treated, and coating + AITC 20 ppm-treated samples by day 18 of storage and remained below the threshold for spoilage (6 log_10_ CFU/cm^2^) throughout storage (Figure 1). The LAB population increased (*P* ≤ 0.05) on the control, coating-only, and coating + AITC 20 ppm samples during storage at 4°C. There were no differences (*P* > 0.05) in the LAB populations between treatments at the end of the 21-d storage period (Figure 2). Aerobic and lactic acid bacteria are common causes of reduced shelf life of perishable food products. A concentration of 25 µL/g of AITC in combination with carrageenan and chitosan has been reported to reduce aerobic bacteria and LAB by at least 1.72 and 0.94 log_10_ CFU/g, respectively, after 21 d of storage (Olaimat et al., 2014). The different efficacies against the 2 types of bacteria could be attributed to the differential effects of AITC against Gram-negative and Gram-positive bacteria (Olaimat et al., 2014). In addition, LAB starter cultures and adventitious LAB in meat have been reported to be more resistant to AITC than *Salmonella* (Olaimat and Holley, 2015), a finding similar to the results of this study that showed LAB to be less sensitive to AITC than aerobic bacteria.

**Figure 3:**
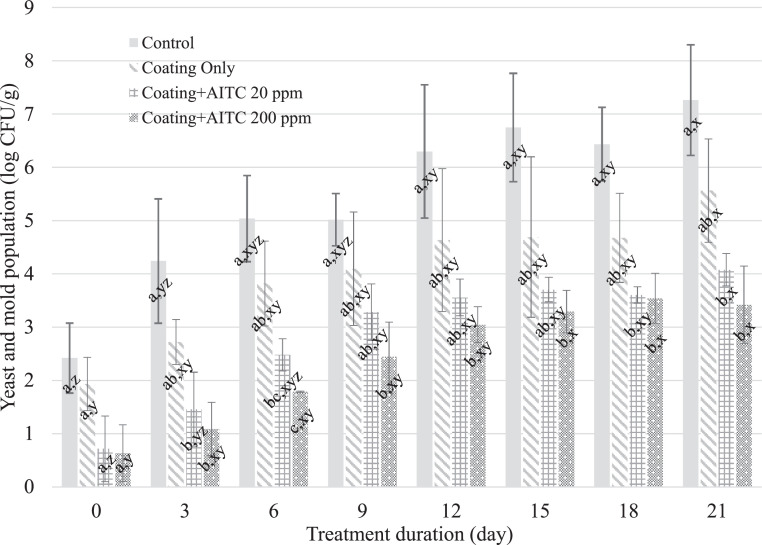
Mean yeast and mold populations on chicken breast treated with no coating, coating only, coating + 20 ppm AITC, and coating + 200 ppm AITC. A–C: Different means within a timepoint are indicated by different superscript letters (*P* ≤ 0.05). X–Z: Different means within a treatment condition are indicated by different superscript letters (*P* ≤ 0.05).

Typical yeast and mold spoilage concentrations are not known; however, in this study, the yeast and mold populations on the chicken breast treated with coating + AITC 20 ppm and coating + AITC 200 ppm were lower compared to the control treatment (*P* ≤ 0.05) by day 18 and this difference persisted until the end of the storage period. Both concentrations of AITC in combination with the coating reduced yeast and mold by at least 3 log_10_ CFU/g at d 21 compared to the untreated control and by >1 log_10_ CFU/g compared to the coating-only treatment (*P* ≤ 0.05) (Figure 3). The antifungal mechanisms of phytochemicals have been reported to include inhibition of cellular membrane biosynthesis, alteration of cellular membrane permeability, and reactivity with proteins thiol-moieties, which cause a reduction in fungal fitness and cell death (Redondo-Blanco et al., 2019). Results from our study suggest that the coating with 20 or 200 ppm of AITC can reduce the number of spoilage microorganisms on chicken breast, which could extend the shelf life of the product.

### Physico-Chemical and Color Properties

**Figure 4:**
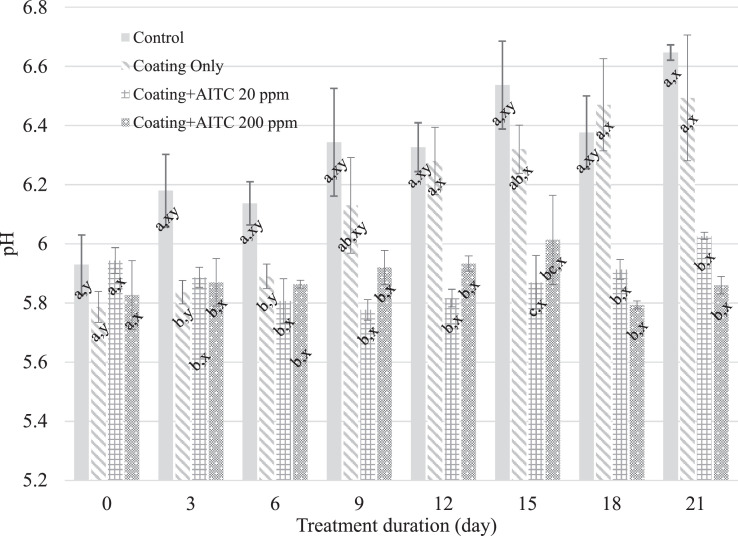
Mean pH of chicken breast treated with no coating, coating only, coating + 20 ppm AITC, and coating + 200 ppm AITC. A–B: Different means within a timepoint are indicated by different superscript letters (*P* ≤ 0.05). X–Y: Different means within a treatment condition are indicated by different superscript letters (*P* ≤ 0.05).

The pH of the untreated chicken breast was higher (*P* ≤ 0.05) than the pH of the chicken breast treated with coating only, coating + AITC 20 ppm, and coating + AITC 200 ppm by d 3 (Figure 4). The pH change of the coating-only-treated chicken breast was delayed through d 6 of storage; however, there was no difference (*P* > 0.05) between the pH of the control and coating-only treatment samples after d 6. The chicken breast samples treated with coating + AITC 20 ppm and coating + AITC 200 ppm showed no significant (*P* > 0.05) pH change by the end of the d 21 storage period, and the pH of the chicken breast for both treatments were lower (*P* ≤ 0.05) than those of the control and coating-only treatment samples at d 21. These results are in agreement with a previous study concluding that white shrimp treated with a chitosan/chito-oligosaccharides/glutathione coating maintained a lower pH than the uncoated control group over 10 d of storage (Wu, 2014). Edible antimicrobial coatings can increase proton concentrations, thereby decreasing the external pH, hence potentially affecting the integrity and permeability of microbial cell membranes and disrupting nutrient transport systems to induce microbial cell death (Lucera et al., 2012).

**Figure 5:**
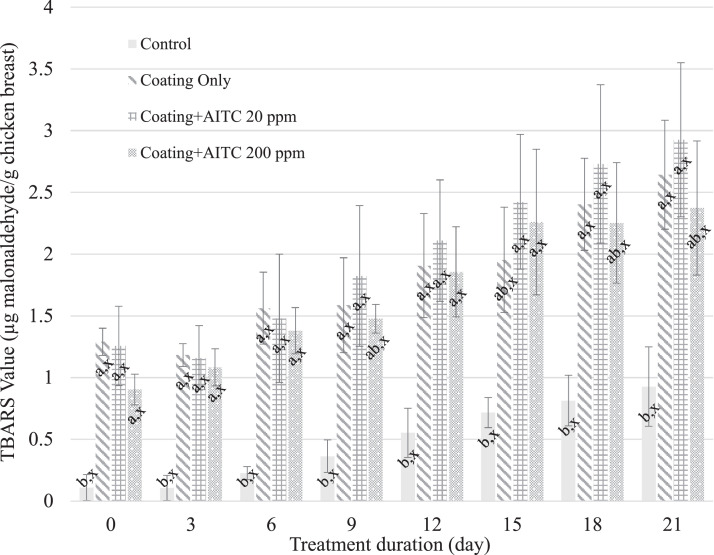
TBARS values expressed as malonaldehyde concentration (µg/g tissue) of chicken breast treated with no coating, coating only, coating + 20 ppm AITC, and coating + 200 ppm AITC. A–B: Different means within a timepoint are indicated by different superscript letters (*P* ≤ 0.05). X: Similar means within a treatment condition are indicated by the same superscript letter (*P* ≤ 0.05).

The carrageenan/chitosan coating alone or in combination with 20 or 200 ppm of AITC resulted in increased (*P* ≤ 0.05) TBARS values (expressed as µg malonaldehyde/g chicken breast) on d 0, compared to the untreated control (Figure 5). Malonaldehyde (**MDA**) is a compound produced during second stage auto-oxidation and is partly responsible for off-flavors and odors that develop during storage (Kang et al., 2013). The TBARS assay values for each experimental treatment group were higher (*P* ≤ 0.05) than those of the control group through d 12 of storage and similar to each other throughout storage. It has been reported that TBARS values increase steadily over storage periods (Azimzadeh and Jahadi, 2018). However, the authors also reported that adding 1% chitosan coating with *L. nobilis* extract resulted in lower TBARS values on coated cashews compared to the control over a 90-d storage period. Another study found that adding 1,000 ppm of AITC to chicken prior to storage for up to 6 d decreased (*P* ≤ 0.05) the TBARS values (Hussein et al., 2019). The difference in the TBARS results from our study compared to the results reported by Hussein et al. (2019) can be attributed to the possibility that the carrageenan/chitosan coating produced byproducts similar to MDA during the TBARS assay that altered the observed values, as the samples with coating, coating + AITC 20 ppm, and coating + AITC 200 ppm did not display the key sensory changes that would be expected to occur from oxidative rancidity.

The chitosan/carrageenan coating alone did not affect (*P* > 0.05) the L*, a*, and b* values of the chicken breast by the end of the 21-d storage period at 4°C, as compared to the untreated control samples (Table 1, Table 2, Table 3). Furthermore, the application of the coating in combination with 20 or 200 ppm treatment of AITC prevented the significant decreases in L* values of the chicken breast that were observed for the untreated control and coating-only samples at d 18 and d 21 of storage. Unlike the control and coating-only-treated samples, the chicken breast treated with 200 ppm of AITC did not (*P* > 0.05) show increased redness over the 21 d of storage. Similarly, the coating applied to the chicken breast in combination with 20 and 200 ppm AITC prevented reduced yellowness over the 21 d of storage compared to the untreated control (*P* > 0.05). In a previous study, no significant changes in color were reported for coating-treated chicken wingettes during a shorter storage period (≤3 d), while the uncoated chicken became more yellow, probably because of the effects of the coating in combination with eugenol over long storage periods (Wagle et al., 2019). Additionally, the lightness of meat is an indicator of a lower pH, a correlation observed for the 20 and 200 ppm AITC-treated samples; pH and lightness values did not change over 21 d of storage (*P* > 0.05). A low pH causes proteins in the muscle to spread out, allowing light to be reflected from the surface of the meat and producing increased lightness (Mir et al., 2017). A carrageenan/chitosan coating with allyl isothiocyanate treatment increased the shelf life of chicken breast stored at 4°C for 21 d, but significant changes in color and rancidity may indicate decreased quality.

**Table 1:** CIE L* values^1^ (lightness) of chicken breast throughout the storage period.

Day	Control	Coating only	Coating + AITC 20 ppm	Coating + AITC 200 ppm
0	62.4 ± 1.5^a^^,^^x^	64.5 ± 0.9^a^^,^^x^	63.8 ± 2.7^a^^,^^x^	68.2 ± 1.5^a^^,^^x^
3	59.1 ± 4.7^b^^,^^x^	63.4 ± 0.8^ab^^,^^xy^	64.6 ± 1.3^ab^^,^^x^	69.3 ± 0.5^a^^,^^x^
6	59.2 ± 2.4^b^^,^^x^	64.5 ± 1.8^ab^^,^^xy^	62.7 ± 2.0^ab^^,^^x^	67.9 ± 1.0^a^^,^^x^
9	57.8 ± 2.1^c^^,^^x^	61.6 ± 2.8^bc^^,^xyz	65.1 ± 1.7^ab^^,^^x^	68.9 ± 1.9^a^^,^^x^
12	56.0 ± 2.3^c^^,^^x^	62.5 ± 0.3^b^^,^xyz	66.2 ± 1.3^ab^^,^^x^	67.9 ± 0.7^a^^,^^x^
15	58.1 ± 2.5^b^^,^^x^	59.6 ± 1.8^ab,^xyz	61.1 ± 4.5^ab^^,^^x^	68.3 ± 1.3^a^^,^^x^
18	56.5 ± 2.6^b^^,^^x^	58.0 ± 1.7^b^^,^^z^	64.8 ± 0.5^a^^,^^x^	67.0 ± 1.6^a^^,^^x^
21	55.7 ± 2.1^b^^,^^x^	55.9 ± 1.7^b^^,^^z^	64.9 ± 2.8^a^^,^^x^	69.8 ± 1.0^a^^,^^x^

1 Mean ± SD.

a–c Significantly different means within a row are indicated by different superscript letters (*P* ≤ 0.05).

x–z Significantly different means within a column are indicated by different superscript letters (*P* ≤ 0.05). Each mean represents 3 replicate experiments per treatment with 2 samples per replicate.

**Table 2:** CIE a* values^1^ (redness) of chicken breast throughout the storage period.

Day	Control	Coating only	Coating + AITC 20 ppm	Coating + AITC 200 ppm
0	−1.2 ± 0.5^a^^,^^y^	−0.8 ± 1.4^a^^,^^y^	−0.7 ± 0.4^a^^,^^y^	−1.4 ± 0.6^a^^,^^y^
3	2.5 ± 0.5^a^^,^^x^	1.7 ± 0.7^ab^^,^^xy^	0.5 ± 0.2^bc^^,^^xy^	0.1 ± 0.2^c,x^
6	1.8 ± 0.4^a^^,^^x^	0.7 ± 1.1^a^^,^^xy^	0.2 ± 0.4^a^^,^^xy^	−0.1 ± 0.7^a^^,^^xy^
9	2.2 ± 1.4^a^^,^^x^	1.2 ± 1.9^a^^,^^xy^	1.5 ± 0.5^a^^,^^x^	0.5 ± 0.5^a^^,x^
12	1.3 ± 0.6^a^^,^^x^	1.1 ± 0.6^a^^,^^xy^	−0.2 ± 1.2^a^^,^^xy^	−0.8 ± 0.8^a^^,^^xy^
15	0.9 ± 0.2^a^^,^^x^	0.7 ± 0.3^a^^,^^xy^	0.7 ± 0.3^ab^^,^^xy^	0.2 ± 0.02^b^^,^^xy^
18	2.3 ± 0.3^a^^,^^x^	1.9 ± 0.8^a^^,^^x^	1.0 ± 0.7^ab^^,^^xy^	0.1 ± 0.0^b^^,x^
21	2.3 ± 0.3^a^^,^^x^	1.9 ± 0.3^a^^,^^x^	1.2 ± 0.7^ab^^,^^x^	−0.1 ± 0.3^b^^,^^xy^

1 Mean ± SD.

a–c Significantly different means within a row are indicated by different superscript letters (*P* ≤ 0.05).

x–y Significantly different means within a column are indicated by different superscript letters (*P* ≤ 0.05). Each mean represents 3 replicate experiments per treatment with 2 samples per replicate.

**Table 3:** CIE b* values^1^ (yellowness) of chicken breast throughout the storage period.

Day	Control	Coating only	Coating + AITC 20 ppm	Coating + AITC 200 ppm
0	7.9 ± 1.0^a^^,^^x^	7.2 ± 1.6^a^^,^^x^	7.0 ± 1.1^a^^,^^x^	8.0 ± 2.6^a^^,^^x^
3	6.1 ± 0.6^a^^,^^xy^	7.3 ± 0.2^a^^,^^x^	7.4 ± 2.0^a^^,^^x^	9.1 ± 1.1^a^^,^^x^
6	4.7 ± 1.1^b^^,^^xy^	5.2 ± 0.5^b,^^x^	6.5 ± 1.1^ab^^,^^x^	8.9 ± 1.0^a^^,^^x^
9	5.1 ± 1.9^a^^,^^xy^	4.7 ± 0.97^a^^,^^x^	8.2 ± 1.7^a^^,^^x^	9.6 ± 1.9^a^^,^^x^
12	4.2 ± 1.4^a^^,^^xy^	6.6 ± 2.1^a^^,^^x^	7.7 ± 1.7^a^^,^^x^	9.2 ± 1.6^a^^,^^x^
15	5.6 ± 2.1^a^^,^^xy^	5.1 ± 2.2^a^^,^^x^	6.4 ± 0.5^a^^,^^x^	8.7 ± 0.6^a^^,^^x^
18	4.9 ± 1.2^b^^,^^xy^	3.2 ± 0.2^b^^,^^x^	9.9 ± 1.5^a^^,^^x^	8.3 ± 0.6^a^^,^^x^
21	3.3 ± 0.6^b^^,^^y^	4.8 ± 1.9^b^^,^^x^	10.4 ± 1.7^a^^,^^x^	10.7 ± 1.8^a^^,^^x^

1 Mean ± SD.

a–b Significantly different means within a row are indicated by different superscript letters (*P* ≤ 0.05).

x–y Significantly different means within a column are indicated by different superscript letters (*P* ≤ 0.05). Each mean represents 3 replicate experiments per treatment with 2 samples per replicate.

This study illustrates the usefulness and effectiveness of coatings applied in conjunction with antimicrobial GRAS compounds to extend the shelf life and delay the microbial spoilage of fresh poultry meat. In the present study, no sensory evaluations of the treated chicken breast were conducted, but other studies have reported reduced acceptability of food products treated with AITC. Further investigation of strategies for minimizing or countering any reduced acceptability could allow for the use of AITC to control spoilage of fresh chicken breast.

## DISCLOSURES

The authors declare that they have no known competing financial interests or personal relationships that could have appeared to influence the work reported in this paper.
